# How the brain heals emotional wounds: the functional neuroanatomy of forgiveness

**DOI:** 10.3389/fnhum.2013.00839

**Published:** 2013-12-09

**Authors:** Emiliano Ricciardi, Giuseppina Rota, Lorenzo Sani, Claudio Gentili, Anna Gaglianese, Mario Guazzelli, Pietro Pietrini

**Affiliations:** ^1^Laboratory of Clinical Biochemistry and Molecular Biology, Department of Surgery, Medical, Molecular, and Critical Area Pathology, University of PisaPisa, Italy; ^2^MRI Lab, Fondazione “G. Monasterio” Regione Toscana/CNRPisa, Italy; ^3^Clinical Psychology Branch, Pisa University HospitalPisa, Italy

**Keywords:** forgiveness, reappraisal, emotional regulation, functional Magnetic Resonance Imaging, effective connectivity

## Abstract

In life, everyone goes through hurtful events caused by significant others: a deceiving friend, a betraying partner, or an unjustly blaming parent. In response to painful emotions, individuals may react with anger, hostility, and the desire for revenge. As an alternative, they may decide to forgive the wrongdoer and relinquish resentment. In the present study, we examined the brain correlates of forgiveness using functional Magnetic Resonance Imaging (fMRI). Healthy participants were induced to imagine social scenarios that described emotionally hurtful events followed by the indication to either forgive the imagined offenders, or harbor a grudge toward them. Subjects rated their imaginative skills, levels of anger, frustration, and/or relief when imagining negative events as well as following forgiveness. Forgiveness was associated with positive emotional states as compared to unforgiveness. Granting forgiveness was associated with activations in a brain network involved in theory of mind, empathy, and the regulation of affect through cognition, which comprised the precuneus, right inferior parietal regions, and the dorsolateral prefrontal cortex. Our results uncovered the neuronal basis of reappraisal-driven forgiveness, and extend extant data on emotional regulation to the resolution of anger and resentment following negative interpersonal events.

## Introduction

Being offended or harmed hurts. Victims of wrongdoing may feel emotional pain, anger, and the desire for revenge toward their offenders, and may also engage in retaliatory behavior. The ability to respond in adaptive manners to adverse events is crucial for the individual social integration. Cognitive evaluation (*appraisal*) plays a key role in shaping the meaning that an interpersonal offense assumes (McCullough, 2001). The way we view an offensive event affects both the valence and intensity of our emotional experience of it. For instance, while generally we feel hatred as victims of a robbery, we may become merciful if we learn that the thief needed unaffordable medical care for his child.

Forgiveness is a cognitive and emotional process that eradicates chronic hostility, rumination, and their adverse effects (Worthington et al., 2007). Psychotherapeutic interventions have capitalized on forgiveness to help patients to adaptively manage anger and negative affect following harmful experiences or interpersonal conflicts (e.g., Fitzgibbons, 1986; Reed and Enright, 2006). Other interventions have shown the utility of forgiveness in solving social and political disputes (Gentilone and Regidor, 1986; Enright et al., 1994).

Negative affect and chronic emotional distress erode health (Hu and Gruber, 2008), alter cardiovascular reactivity (Holt-Lunstad et al., 2008), impoverish sleep quality (Stoia-Caraballo et al., 2008), stimulate the production of stress-related hormones, such as cortisol (Berry and Worthington, 2001), being associated over time with the development of clinical conditions such as depression (e.g., Nolen-Hoeksema and Morrow, 1991). Conversely, forgiveness promotes wellbeing (see Worthington et al., 2007 for a review), cardiovascular health (Lawler et al., 2005), and may increase survival rates (Chida and Steptoe, 2008). Specifically, *trait forgiveness* (i.e., a constant attitude to forgive) is associated with a diminished recourse to medications and alcohol, and *state forgiveness* (i.e., a situation-contingent forgiveness) with reduced heart rate and physical symptoms (Lawler-Row et al., 2008). This literature converges suggesting that forgiveness represents a positive, “healthy” strategy for the individual to overcome a situation that otherwise would be a major source of stress from a psychological and neurobiological point of view.

In spite of its relevance in individual and social context, little is still known of the neural basis of interpersonal forgiveness. Farrow et al. (2001) showed activation in prefrontal and posterior cingulate cortical areas while volunteers judged to what extent different crimes (e.g., stealing and personal assault) could be held forgivable given the circumstances under which they took place. Also, a number of studies have addressed the neural mechanisms underlying exculpation (e.g., Farrow et al., 2005; Young and Saxe, 2009; Hayashi et al., 2010). However, to date, the functional neuroanatomy of granting interpersonal forgiveness or unforgiveness in response to personal offenses remains unexplored. The fact that forgiving is a healthy resolution of the problems caused by injuries suggests that this process might have evolved as a favorable response that promotes human survival. Thus, identifying its neural correlates is important in order to clarify which brain areas contribute to such an important biological function, i.e., the restoration of the individual's biological and mental homeostatic equilibrium. In turn, a deeper understanding of the biological bases of a mental process that naturally allows the individual to solve a stressful situation may provide a rationale for the therapeutic use of forgiveness.

In the present study, we used functional Magnetic Resonance Imaging (fMRI) to investigate regional brain activity and cortical effective connectivity associated with forgiveness and unforgiveness. Volunteers were engaged in script-driven mental imagery of personal offense, and subsequently instructed to either grant forgiveness to their imagined offender, or to fuel resentment and/or imagine revenge. We assumed that forgiveness would re-establish the individual emotional balance after an interpersonal hurtful event and lead to subjective relief (Worthington, 2006). We hypothesized that this homeostatic process would engage activation in the dorsolateral prefrontal cortex (DLPFC) and the anterior cingulate cortex (ACC), which are part of the brain network that supports the cognitive regulation of emotion, and have been consistently associated with the decrease of negative affect mediated by reappraisal strategies (Ochsner and Gross, 2005). Also, we expected that in order to forgive, volunteers would need to inhibit spontaneous aggressive reactions in response to personal offenses, and that this process would recruit prefrontal cortical areas, which are known to be involved in the modulation of aggressive behavior (Pietrini et al., 2000; Chikazoe, 2010).

## Methods

### Participants

Ten healthy volunteers (5 females) with 19.2 ± 2.06 years (mean ± *SD*) of education, aged 26 ± 2 years (mean ± *SD*), and with excellent imagery abilities [Gordon Visual Imagery Test, (Richardson, 1969), score = 23.7 ± 0.5 (mean ± *SD*), minimum score = 0 (*worst imagery*), maximum score = 24 (*best*); Betts Questionnaire of Mental Imagery (Sheehan, 1967) vividness score = 79 ± 13.5, score range 35 (*best imagery*) to 245 (*worst*)] were recruited. Participants were right-handed and had no history of any medical, neurological, or psychiatric condition that could affect brain function. None of them was on medication at the time of their enrolment in the study. Volunteers had normal or corrected-to-normal visual acuity, and gave their written informed consent to the study. The Ethics Committee of the University of Pisa approved the study [Protocol n. 1616/2003 03-FMRI-001].

### Experimental paradigm

While in the MRI scanner, subjects were presented with narrative scenarios devised to evoke three consecutive emotional conditions: a pre-hurtful condition, a hurtful condition, and a forgiving or an unforgiving response. Scenarios for the hurtful condition consisted of short stories adapted from the Willingness to Forgive Scale (DeShea, 2003) and were selected among those eliciting the strongest emotional responses based on a rating by an independent sample [*N* = 97, 50 females; mean age ± *SD*: 32 ± 10 years]. We relied on a global pool of 39 hurtful scenarios. Each scenario began with a neutral social scene (*pre-hurtful condition*), and then led to a hurtful event (*hurtful condition*). The indication to forgive or to unforgive the offender were presented in a randomized way for all volunteers (the ratio being: 50% forgiveness requests and 50% unforgiveness requests) for a total of 15 scenarios of forgiveness and 15 scenarios of unforgiveness. The association between a scenario and a specific response (i.e., forgiving or unforgiving) was randomly assigned in each subject, so that a scenario could have two different outcomes in two distinct participants. Indications to forgive were accompanied by one among the following four types of reappraisal-instructions selected on the basis of each specific scenario: (1) statements explaining that emotional distress played an important role in determining the wrongdoers' behavior; (2) justifications of the offender's behavior; (3) statements explaining that the victim him/herself could share some responsibility for the negative event, or (4) positive re-evaluations of the consequences of negative events. Indications for unforgiveness were associated with (1) statements emphasizing the victim's resentment and/or (2) incitements to seek revenge (See Table 1 for examples). Before the beginning of the fMRI scanning session, volunteers were clearly instructed to follow the indications that would be provided with each scenario. The selection of strategies was based on prior literature linking positive reappraisal and empathy toward a wrongdoer to forgiveness (Shapiro, 1991; Fincham, 2000; Batson and Ahmad, 2001; McCullough, 2001). For each scenario, forgiveness/unforgiveness requests were randomized across subjects. To increase imageability, scenarios were tailored on each participant (e.g., referring to a sister in case the volunteer had no brother). Also, scenarios that subjects had actually experienced in their life were discarded. Written descriptions for each scenario were projected on a screen. Each participant was debriefed after the whole experiment.

**Table 1:** **Exemplar narrative scenario**.

**Pre-hurtful condition**
Now you have to imagine that you're having a meeting with your boss and colleagues. Your boss is planning the future work of the company.
**Hurtful condition**
Unexpectedly, he criticizes your job, pointing out your lack of professional skills. He fires you, telling that you must leave the working-place by the day after.
**Forgiveness**
Now imagine to forgive your boss knowing that you never gave your best at work.
**Unforgiveness**
Now imagine not to forgive your boss, to harbor a grudge toward him and think how to revenge in the best way.

The whole experimental paradigm consisted of ten runs, each of which comprised three self-paced blocks separated by a variable inter-stimuli interval (range = 21–30 s). In each block, subjects were given a maximum of 45 s to go through the three screens evoking the intended emotional reaction for each scenario. Volunteers were allowed to read and imagine each scenario at their own pace, and advance through the screens by button pressing. At the end of each run, subjects rated their ability to elicit a detailed mental representation, the intensity of the evoked emotional response for each scenario and the degree of relief that they felt after forgiving by using a self-rating Likert-type scale ranging from 1 (not at all) to 5 (extremely) (Table 2).

**Table 2:** **Self-rating questionnaire on imaginative capabilities**.

1. How clear and vivid was the picture of your imagined scene?
2. How angry and frustrated were you in the imagined context?
3. How easily could you imagine to not forgive and to think about revenge?
4. How easily could you imagine forgiving?
5. How much did you feel better after forgiving?

### Behavioral data analyses

We tested the hypothesis that forgiveness would affect emotional state by re-establishing emotional balance (thus, leading to subjective relief) by computing a correlation analysis between the capacity to grant forgiveness, as rated by the subjects, and levels of subjective relief (linear regression analysis, Bonferroni corrected). To test the hypothesis that volunteers would need to inhibit spontaneous aggressive reactions to forgive personal offenses we ran a correlation analysis between levels of anger and frustration for hurtful scenarios and the subsequent ability to enact unforgiveness (linear regression analysis, Bonferroni corrected).

### fMRI scanning parameters and data analysis

Subjects were instructed not to move and had their head immobilized within the head coil with foam rubbers. We acquired Echo-Planar Images (*TR* = 3000 ms, 26 slices, 5-mm-thick axial images, FOV = 24 cm, *TE* = 30 ms, flip angle = 90°, voxel size = 3.75 mm × 3.75 mm × 5 mm) sensitive to the BOLD signal on a 1.5 Tesla whole body scanner (General Electric, Milwaukee, WI). High-resolution T1-weighted spoiled gradient recall images (124 slices, 1.2-mm-thick sagittal images, FOV = 24 cm) were obtained for each subject as an anatomical reference. AFNI and SUMA packages and related software plugins (http://afni.nimh.nih.gov/afni) were used to analyze functional imaging data. All volumes from the different runs were concatenated and coregistered (*3dvolreg*), temporally aligned (*3dTshift*), and spatially smoothed (FWHM = of 4.5 mm). Individual run data were normalized by calculating the mean intensity value for each voxel, and by dividing the value within each voxel by its mean to estimate the percent signal change at each time point.

A multiple regression was performed to identify brain responses in all the conditions. We modeled pre-hurtful, hurtful, and the forgiving or unforgiving conditions by using four regressors of interest. The pre-hurtful and hurtful conditions were modeled according to each individual pace timing. Brain activation elicited during forgiveness or unforgiveness responses was estimated using a floating window (length = 15 s; i.e., five EPI images), an approach that previously proved effective to investigate complex and temporally variable cognitive/emotional processes (e.g., Greene et al., 2001). The “floating window” technique for data analysis is appropriate for our experimental paradigm as it required participants to deal with a complex and extended in time affective process at their own pace. The floating window surrounded the point of button press during the passage from the hurtful scenario to the response (forgiving/unforgiving) outcome (thus, encompassing one EPI image during button press and four EPI images after the button press, to include the hemodynamic delay of the neural response). We excluded from the analysis all the scenarios for which volunteers were unable to elicit a detailed mental representation based on their post-scanning self-ratings. Similarly, trials through which participants went too quickly (<5 s) or too slowly (>45 s) were excluded from further analysis.

The six movement parameters derived from the volume registration and the polynomial regressors accounting for baseline shifts and linear/quadratic drifts in each scan series were included as regressors of no interest. Individual unthresholded magnitude response and statistical maps of each condition of interest were transformed into the Talairach and Tournoux Atlas coordinate system, and resampled into 1 mm^3^ voxels for group analyses. Activations were anatomically localized on the group-averaged Talairach-transformed T1-weighted images and visualized using SUMA surface templates.

We used the unthresholded-weights of each condition of interest to construct T contrasts and identify both significant patterns of neural response for each condition as compared to rest (equivalent to one-sample group *T*-tests) and significant differences between conditions (equivalent to paired *T*-tests). Considered comparisons were: (1) hurtful vs. pre-hurtful condition; this contrast was run to determine brain regions whose activation was elicited by imagining hurtful scenarios as compared to neutral ones; (2) [response conditions] vs. [pre-hurtful + hurtful conditions]; this contrast was computed in order to determine brain networks involved in the regulation of emotional responsiveness as compared to preparatory neutral and hurtful scenarios; (3) forgiveness vs. unforgiveness; this contrast was computed to identify brain regions differentially involved in forgiveness as compared to unforgiveness. Contrast (3) was run with the aim of testing the hypotheses that forgiveness would engage activation in the DLPFC and the ACC, (as these brain areas are engaged during the cognitive regulation of emotion that are mediated by reappraisal) and that an inhibition of spontaneous aggressive reactions in response to personal offenses recruiting prefrontal areas was needed to enact forgiveness. The correction for multiple comparisons across the whole brain was conducted using MonteCarlo simulations run via *3dClustSim* in AFNI with a voxel-wise threshold of 0.01, that resulted in a minimum cluster volume of 492 μ L, with a cluster connection radius of 1.73 mm for a corrected *p*-value of 0.05 at cluster level.

#### Effective connectivity analysis

A multivariate autoregressive analysis was used to search for differences between task-related networks during forgiveness and unforgiveness. We examined effective connectivity between brain regions responsive during the two conditions by computing a pair-wise Granger Causality (GC) analysis in frequency based on a vector autoregressive model. The ROIs included the activated clusters that were found by contrasting the forgiveness and unforgiveness conditions, as well as by contrasting the forgiveness and unforgiveness conditions with the pre-hurtful and hurtful conditions (voxelwise *p* < 0.01, cluster-level *p* < 0.05, as calculated via *3dClustSim* in AFNI). We selected and concatenated all “task-related” fMRI time-series corresponding to each ROI. Afterwards, we computed a pair wise GC analysis in frequency. The VAR model was fitted separately for each time-series, obtaining a GC spectrum for each pair of ROIs time-series of each subject. We applied a low-pass filter (range = 0–0.12 Hz) to the pre-processed functional dataset to remove artifacts due to cardiac and respiratory pulsatility. As in Deshpande et al. (2009), we summed all frequency components of each relative spectrum, obtaining a single GC value for each connection in each subject. We created a bootstrapped distribution based on a 1000 resamples of estimated autoregressive coefficients that describe the ROIs time-series. Afterwards, we compared GC values to this distribution, testing the null hypothesis of no causality between two ROIs (Kolmogorov-Smirnov, *p* < 0.05). We computed group analysis maps (*p* < 0.05), evaluating a single *p*-value for each connection, and combining *p*-values from individual subjects using the Fisher's method. Furthermore, we selected each subject's GC maps during forgiveness and we determined which GC map value correlated with the ratings related to the ability to forgive and level of relief following forgiveness, respectively. Finally, we correlated each subject CG maps values during unforgiveness with the behavioral ratings related to the ability to feel resentment and revenge.

## Results

### Behavioral data

All volunteers confirmed that they used the strategies for achieving forgiveness and unforgiveness that had been indicated time by time with each scenario. None of them raised any issue concerning difficulties in using those cognitive strategies for the imagery task. All subjects were able to mentally imagine the scenarios presented during the distinct experimental conditions. Five percent of all scenarios were excluded from fMRI data analysis due to poor imageability (<2 scores) and reading times (<5 s and >45 s). The highest number of scenarios excluded for each subject was *N* = 4 (mean = 1.3; *SD* = 1.7). Scores for the self-rating Likert-type scales were: ability to imagine the intended scenarios = 3.64 ± 0.52; level of anger and frustration during the imagination of hurtful scenarios = 3.15 ± 0.71; ability to forgive = 3.12 ± 0.73; ability to feel resentment and revenge = 3.08 ± 0.76; level of relief following forgiveness = 2.60 ± 0.67. The ability to enact forgiveness or unforgiveness did not differ significantly (paired *t*-test, *p* > 0.05, *N* = 10). The capacity to grant forgiveness strongly correlated with subjective relief (linear regression analysis, *r* = 0.94, *p* < 0.01 one-tailed, Bonferroni corrected, *N* = 10). The ability to enact unforgiveness correlated with the vividness with which subjects were able to imagine previous hurtful scenarios (linear regression analysis, *r* = 0.90, *p* < 0.01 one-tailed, Bonferroni corrected, *N* = 10) and feel anger and frustration (linear regression analysis, *r* = 0.93, *p* < 0.01 one-tailed, Bonferroni corrected, *N* = 10).

Response times did not differ for the pre-hurtful and hurtful conditions (mean reaction ± *SD* times for pre-hurtful = 11.8 ± 4.4 s and hurtful = 14.9 ± 4.7 s; *p* = ns).

### Functional brain responses

#### Hurtful vs. pre-hurtful condition

This contrast determines brain regions whose activation was specifically prompted by imagining a hurtful scenario as compared to a neutral “baseline” condition. A significantly greater recruitment during hurtful as compared to pre-hurtful scenarios was found in right dorsal-medial prefrontal areas, and left temporo-parietal junction, medial temporal gyrus (MTG), and precuneus.

#### Response condition vs. pre-hurtful and hurtful conditions

**Figure 1:**
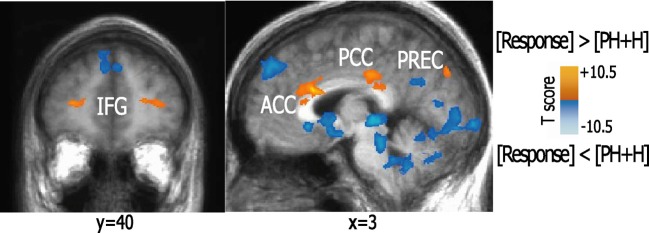
**Activation patterns elicited during the response condition as compared to pre-hurtful and hurtful conditions.** The figure shows brain areas active during forgiveness and unforgiveness as compared to pre-hurtful and hurtful scenarios. Statistical parametric maps (*p* < 0.01 corrected) of activations are visualized on a brain template. Abbreviations: IFG, inferior frontal gyrus; ACC, anterior cingulate cortex; PCC, posterior cingulate cortex; PREC, precuneus.

When examining the brain networks involved in the regulation of emotional responsiveness as compared to preparatory neutral and hurtful scenarios, we found a higher activation in ACC and posterior cingulate cortex (PCC), parahippocampal/occipital cortex (OCC), right precuneus, right inferior parietal cortex (BA40), left anterior middle frontal (BA11/10), and right superior-medial prefrontal (BA6) areas (Figure 1).

In contrast, pre-hurtful, and hurtful conditions showed a greater recruitment of bilateral striate and extrastriate visual cortex, sensorimotor fronto-parietal areas, supplementary motor area, anterior MTG and medial prefrontal areas, left temporo-parietal, premotor areas, subgenual cingulate cortex, thalami and cerebellum.

#### Forgiveness vs. unforgiveness

**Figure 2:**
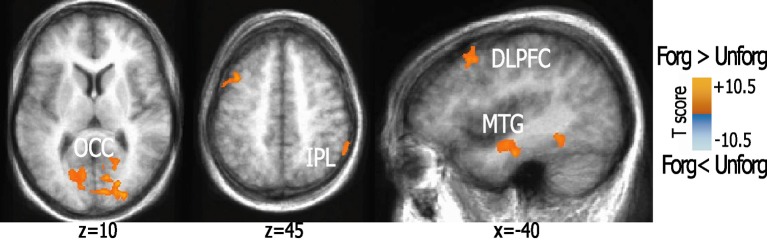
**Activation patterns elicited during forgiveness as compared to unforgiveness.** The figure shows brain areas active while subjects granted forgiveness to imagined offenders. Statistical parametric maps (*p* < 0.01, corrected) of activation elicited by forgiveness as compared to unforgiveness-scenarios are visualized on a brain template. Abbreviations: DLPFC, dorsolateral prefrontal cortex; IPL, inferior parietal lobule; OCC, middle occipital cortex; MTG, anterior medial temporal gyrus.

This comparison showed activation in left DLPFC (BA8) and right inferior parietal lobule (IPL, BA40) as well as in bilateral MTG (BA20). Additional activations in extrastriate regions, bilateral cuneus, fusiform, and anterior parahippocampal regions, and PCC clusters were also found. For details, see Figure 2.

#### Effective connectivity analysis

**Figure 3:**
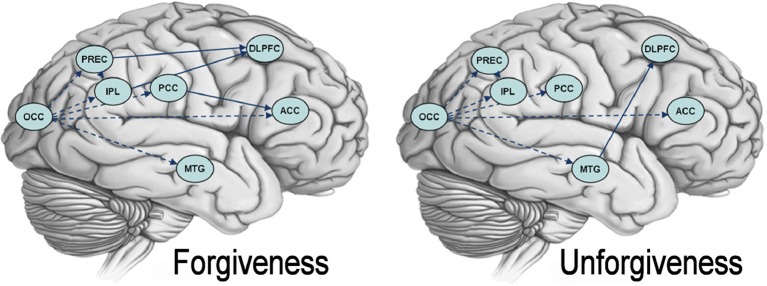
**Granger Causality maps for forgiveness and unforgiveness.** The picture depicts brain networks causally connected during forgiveness (on the left) and unforgiveness (on the right). Dashed lines show causal connections common to both conditions. Abbreviations: DLPFC, dorsolateral prefrontal cortex; ACC, anterior cingulate cortex; PCC, posterior cingulate cortex; PREC, precuneus; IPL, inferior parietal lobule; OCC, occipital cortex; MTG, medial temporal gyrus.

As detailed before, a multivariate autoregressive analysis was used to search for differences between task-related networks during forgiveness and unforgiveness. During both forgiveness and unforgiveness, patterns of activation in the OCC predicted subsequent activation in the precuneus, the IPL, the ACC, the PCC, and the MTG. Also, activation in the precuneus predicted subsequent activation in the IPL. During forgiveness, BOLD responses in the precuneus and the IPL predicted activation in the DLPFC and BOLD responses in the PCC influenced ACC's activation. During unforgiveness, activation patterns in the MTG influenced DLPFC activation. See Figure 3 for group maps of the effective connectivity during the forgiveness and unforgiveness responses.

The strength of the connection between precuneus and inferior parietal lobule during forgiveness significantly correlated with the ratings of the levels of relief as experienced by our volunteers (*p* = 0.034, *r* = 0.67). We did not find significant correlations between GC values during forgiveness and the vividness of imagination and between the GC values during unforgiveness and the ratings related to the ability to feel resentment and revenge.

## Discussion

In the present study, we investigated brain functional activity associated with granting forgiveness and unforgiveness following a hurtful event. Volunteers were engaged in script-driven mental imagery of interpersonal wrongdoings resulting in a hurtful condition and were instructed either to forgive or to feel resentment and think about revenge toward imagined offenders. We accompanied each forgiveness request with an adjunctive explanation modulating the victim's perception of wrongdoings in order to lead to a benevolent reappraisal. Specifically, we presented positive re-evaluations of the negative events and their consequences, explanations of the offender's motivations and considerations about the role of the victims themselves, or descriptions of the wrongdoers' distressed emotional state. Conversely, unforgiveness requests were associated with statements emphasizing the victim resentment and invitations to respond to the misconduct with revenge. We hypothesized that reappraisal-driven forgiveness would recruit brain areas involved in the cognitive regulation of emotional responses and would be associated with subjective relief.

Self-rating scores indicated that volunteers were able to vividly imagine each experimental condition of interest and experience a strong emotional response. Consistently with our hypotheses, the ability to forgive imaginal offenders strongly correlated with subjective relief, and was selectively associated with neural activation in a causally connected brain network comprising the DLPFC, the precuneus, and the IPL.

### Reappraisal processes

The DLPFC is recruited when individuals use cognitive strategies to regulate emotional responsiveness (Ochsner and Gross, 2005). This brain area is part of a cognitive circuit of top-down control that mediates the volitional suppression of negative affect (Ochsner and Gross, 2005) and sadness (Lévesque et al., 2003). Cognition is essential for emotions to be generated (Schachter and Singer, 1962) and plays a crucial role in emotional regulation (Gross, 2002) and psychotherapeutic effects (Beauregard, 2007). According to *appraisal theory*, the way a person thinks about a certain experience or event shapes his emotional connection to it (Lazarus, 1991). The ability to reappraise negative events in positive terms, such as by re-interpreting the motivations of the offender in a benevolent manner (McCullough, 2001) is a key step in the process to forgive a wrongdoer. Thinking of a negative event as being not too bad (Shapiro, 1991), or that the wrongdoers were not directly responsible for their act (Fincham, 2000), that they did not intend to harm (Frijda, 1986; Shapiro, 1991), or that they were instead motivated by altruistic reasons (Lindskold and Walters, 1983), reduces feelings of injustice, punitiveness, and unforgiveness in response to interpersonal transgressions. Thus, operating a benevolent/positive evaluation of the meaning of emotionally charged situations modifies the significance of these events, paving the way for forgiveness to take place. Neural activation in the DLPFC during forgiveness is consistent with our hypotheses and with the present findings.

We did not report activations in ACC and PFC areas other than DLPFC as they did not reach significance. This result may be due to the fact that reappraisal strategies were only one of the sets of strategies that were employed for volunteers to enact forgiveness. One may expect that a higher number of items containing more selectively reappraisal instructions might have enhanced activation in additional brain sites. Low power might also be the reason for our selective findings. Also, activation in the DLPFC during forgiveness may reflect response inhibition processes (Asahi et al., 2004) that were plausibly engaged during the process of forgiving a wrongdoer.

Our results extend previous accounts on the role of the DLPFC in reappraisal by showing that this area is the crucial node of a network of brain areas causally connected during forgiveness, which comprises the precuneus and the IPL.

### Perspective taking processes and empathy

The precuneus is recruited when subjects are instructed to assume the perspective of a third-person (Cavanna and Trimble, 2006). Mental perspective-taking is essential for making sense of the mind of others, and relies on the construction of an internal representation of their mental states (*theory of mind*, Leslie, 1987). In our study, mental perspective-taking processes were required for volunteers to make sense of the motivations and reasons underlying the offender's behavior during both forgiveness and unforgiveness. Consistently, we observed activation in the precuneus during both processes. Thus, the recruitment of this brain area likely reflects perspective-taking, a process that may foster empathy (Oswald, 1996). Empathy toward a wrongdoer extinguishes retaliatory behavior, even when it would lead to an economic gain for the victim (Batson and Ahmad, 2001). Presumably, a similar process takes place during forgiveness. Taking a broader view on the offenders' personality rather than emphasizing the gravity of their act only, evaluating the factors that might have led to their behavior, as well as being able to see them as similar to oneself, are examples of cognitive strategies that help feeling empathy, making it possible to forgive (North, 1998).

The IPL is recruited when subjects attribute emotional states to others and assume an empathetic attitude toward their suffering (Saxe and Wexler, 2005; Nummenmaa et al., 2008). In our study, activation in this site was selective for forgiveness and is consistent with the idea that volunteers operated an emotional attribution, a process that helps empathize with their offender. A link between empathy and forgiveness has already been reported (McCullough et al., 1997; Worthington, 1998; Zechmeister and Romero, 2002). Our functional connectivity results provide a neurobiological counterpart to these findings by showing a selective causal interaction between IPL, precuneus and DLPFC during forgiveness. The causal interplay of brain sites responsible for theory of mind, empathy and the cognitive regulation of emotions, indicates that being able to see a misconduct with the eyes of the offenders is necessary for a victim to empathize with them, and that both these processes are useful to reconsider the evaluation of negative events (emotionally and cognitively-driven reappraisal) in positive terms. Also, for both forgiveness and unforgiveness, GC results showed that activation in OCC regions predicted subsequent activation in PREC, IPL, PCC, ACC, and MTG. This result suggests that visual imagery components might have triggered subsequent engagement of brain regions involved in perspective taking processes and empathy resulting in the engagement of the DLPFC during forgiveness. The fact that the strength of the connection between precuneus and inferior parietal lobule and levels of relief were correlated suggests that a network of brain regions, rather than a unique brain site, is responsible for triggering this complex cognitive and emotional process.

Also, this data is interesting as it points to a specific involvement of the link between the precuneus and the inferior parietal lobule in the subjective feeling of relief. Thus, perspective taking abilities predict the ability to empathize with a wrongdoer and together these elements are effective in inducing positive affective states. Functional connectivity in these specific brain sites may contribute to the role of forgiveness as a wellbeing promoting mechanism.

### Decision-making and inhibition of spontaneous aggressive reactions

Inferior frontal areas and the ACC were recruited during both forgiveness and unforgiveness. Activation in these areas likely reflects decision-making processes about wrongdoings and evaluation of actions to enact and their long-term consequences (Carter et al., 2000; Addis et al., 2007). The involvement of the ACC may reflect the homeostatic function of decision-making processes (Paulus, 2007) that allows the individual to re-establish the subjective emotional balance after a hurtful interpersonal event. Nevertheless, activation of this brain site has also been consistently associated with emotional regulation processes mediated by reappraisal strategies (Ochsner and Gross, 2005) as well as with tasks involving attention, cognitive control, and performance monitoring (e.g., Weissman et al., 2005; Walsh et al., 2011). Thus, its activation may reflect multiple intervening cognitive processes that were engaged during decision making underlying both forgiveness and unforgiveness.

Self-ratings on emotional states indicate that levels of anger and frustration experienced when imagining negative events correlated with the subsequent ability to feel resentment. This result suggests that victims spontaneously develop aggressive reactions and hostility in response to unfair events, which then are likely to lead to resentment and the desire of revenge. Typically, people holding a grudge rehearse memories of past hurtful events (Baumeister et al., 1998). This condition, known as rumination, is believed to amplify and perpetuate thoughts of hostility and sadness (Trask and Sigmon, 1999; Vickers and Vogeltanz-Holm, 2003), which over time are associated with the development of clinical conditions including depression (Nolen-Hoeksema and Morrow, 1991). Thus, the capacity to forgive an offender requires the inhibition of spontaneous aggressive responses. Also, relief-levels following forgiveness strongly correlated with the capacity to forgive. Taken together, these results indicate that benevolent reappraisal is effective in suppressing spontaneous aggressive reactions in response to unfair actions. The present results expand current literature on emotion regulation.

Here, we employed cognitive strategies to lead volunteers to forgive or feel anger and the desire to get revenge toward a wrongdoer. Nevertheless, emotional reappraisal was not the only variable that we investigated. Viewing events from the offender's point of view (i.e., perspective taking abilities) and the ability to emotionally identify oneself with the wrongdoer were also considered as important components of forgiveness. The novelty of our study consists in evaluating all these three aspects (i.e., cognitive appraisal, perspective taking abilities, and empathetic concern) as main triggers of the forgiveness response.

### Limitations of the study

In the present study we relied on a script-driven mental imagery paradigm, and this might represent a limitation for the extensibility of the present results to real-life situations. However, mental imagery paradigms have proven effective for the study of processes that for ethical and/or technical constrains cannot be addressed in a naturalistic fashion, such as unrestrained aggressiveness (Pietrini et al., 2000). This paradigm allowed us to control for multiple variables that intervene in real-life situations (e.g., social circumstances under which personal offenses take place, gravity of the consequences of interpersonal offenses, etc.) in an experimentally controlled way. The fact that volunteers were instructed to enact forgiveness by relying on reappraisal strategies may also represent a bias. On the other hand, by relying on a set of pre-defined cognitive strategies, we were able to control for multiple intervening cognitive processes that otherwise could have been engaged differentially by each participant, thus, affecting the interpretation of the results. Also, reappraisal-driven forgiveness affected the subjects' emotional state positively, thus, indicating that reappraisal effectively changes the emotional experience of negative events.

As a matter of fact, cognitive strategies that may be useful for dealing with negative events may vary from individual to individual and also within each person from time to time. While forgiveness has been proposed to be effective in dealing with wrongdoings in a positive and constructive manner (Worthington et al., 2007), other strategies such as distancing may be used for dealing with negative events. In our study, we did not test for other strategies useful for confronting negative events rather than the ones previously associated to forgiveness for constraints due to length and complexity of the experimental design. A more systematic study which includes further cognitive strategies should be carried out in the future to obtain a more comprehensive view of the neural underpinnings of different manners for dealing with negative events.

In the present study, fMRI data was acquired from a relatively small sample of subjects. Our results are preliminary and future investigations are warranted to replicate the present findings in a greater number of volunteers.

## Conclusions

In summary, our study explored for the first time the neural correlates of forgiveness. We observed a link between forgiveness and subjective relief, which supports its use in therapeutic settings as an aid for the promotion of mental health. We observed activation in a brain cortical network responsible for perspective taking processes, appraisal and empathy, suggesting that these processes may play an important role in the adaptive extinction of negative affect and prevention of potential aggressive and socially unacceptable behavior.

## Conflict of interest statement

The authors declare that the research was conducted in the absence of any commercial or financial relationships that could be construed as a potential conflict of interest.
