# Supplementary material for: How the brain heals emotional wounds: the functional neuroanatomy of forgiveness
Source: Front Hum Neurosci. 2013 Dec 9;7:839. doi: 10.3389/fnhum.2013.00839 (PMC3856773; doi:10.3389/fnhum.2013.00839)
Supplement: Supplementary file 1 [file Presentation1.PDF]

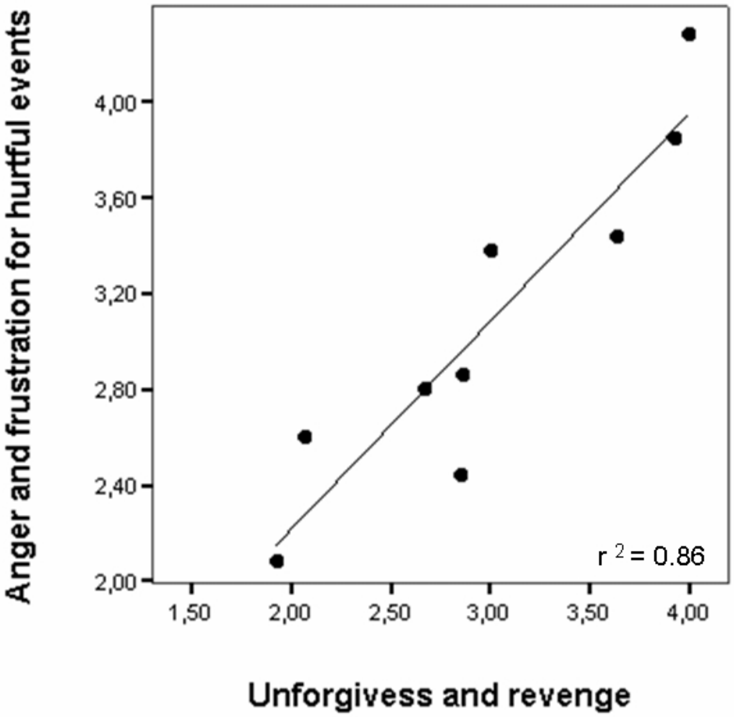
Anger and frustration for hurtful events
4,00
3,60
3,20
2,80
2,40
2,00
1,50
2,00
2,50
3,00
3,50
4,00
r 2 = 0.86
Unforgivess and revenge

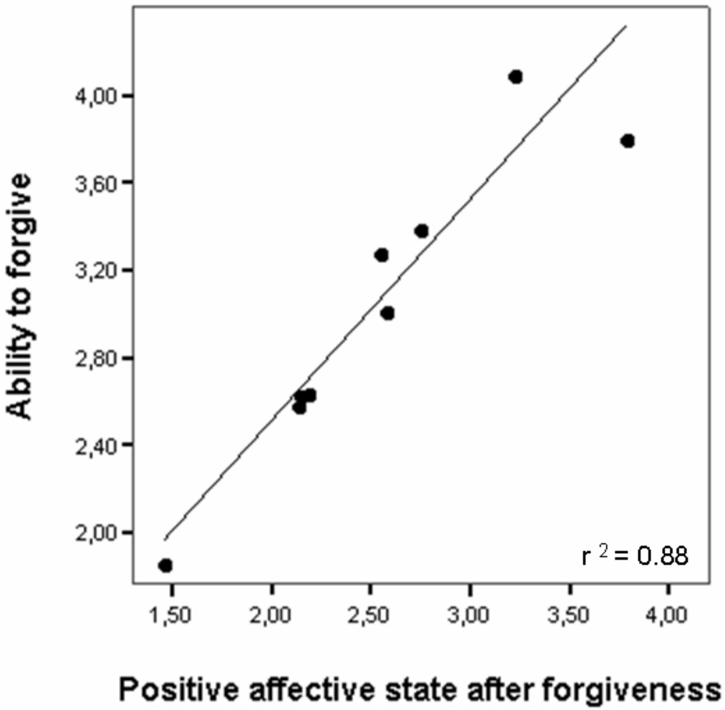
Ability to forgive
4,00
3,60
3,20
2,80
2,40
2,00
1,50
2,00
2,50
3,00
3,50
4,00
r 2 = 0.88
Positive affective state after forgiveness
