# Supplementary material for: How the brain heals emotional wounds: the functional neuroanatomy of forgiveness
Source: Front Hum Neurosci. 2013 Dec 9;7:839. doi: 10.3389/fnhum.2013.00839 (PMC3856773; doi:10.3389/fnhum.2013.00839)
Supplement: Supplementary file 2 [file 48075__Data_Sheet_1.DOC]

**Supplementary** **Table 1A.** The table lists brain areas, Brodmann’s areas, Talairach coordinates and *t* scores for all loci of activation. Abbreviations: HS = Hemisphere, BA = Brodmann’s areas, L = left hemisphere, R = right hemisphere. In Table 1A, brain areas have been descriptively characterized by their peak values of gray matter voxels (accordingly to the Talairach Daemon Atlas) in their respective T scores maps (separation distance > 20 mm).

**Supplementary** **Table 1A. Brain loci of activation for the conditions of interest**

| **Brain Area** | | **HS** | **BA** | **Coordinates** | *t* **score** |
| --- | --- | --- | --- | --- | --- |
| **Pre-hurtful > Rest** | | | | | |
| Middle Occipital Gyrus  Middle Occipital Gyrus  Lingual Gyrus  Inferior Occipital Gyrus  Fusiform Gyrus  Fusiform Gyrus  Thalamus  Parahippocampal Gyrus  Cerebellum  Medial Frontal Gyrus  Medial Frontal Gyrus  Anterior Cingulate Cortex  Posterior Cingulate Cortex  Precuneus  Precuneus  Middle Temporal Gyrus  Middle Temporal Gyrus  Supramarginal Gyrus  Inferior Frontal Gyrus  Inferior Parietal Lobe  Caudate  Precentral Gyrus  Superior Temporal Gyrus  Superior Temporal Gyrus | | R  L  R  L  R  L  R  R  L  L  L  R  R  R  L  R  L  L  L  L  L  L  L  R | 18  18  17  19  19  36  6  10  25  29  31  23  22  22  40  9  40  6  22  38 | 15 -99 6  -24 -98 6  11 -93 -6  -33 -87 -14  25 -67 -10  -34 -67 -10  21 -26 0  25 -28 -16  -6 -73 -17  -6 -7 57  -3 41 -7  2 10 -4  2 -49 12  -3 -65 18  2 -61 20  53 -7 -7  -55 -8 -7  -55 -48 26  -52 10 28  -49 -44 28  -5 4 -2  -58 1 26  -58 8 -3  52 -1 -7 | 3.38  4.46  3.39  3.97  3.93  4.39  5.22  5.09  4.48  4.25  4.16  4.34  3.91  4.06  4.83  6.91  7.51  3.35  2.26  4.13  7.32  3.86  4.83  4.57 |
| **Hurtful > Rest** | | | | | |
| Cuneus  Lingual Gyrus  Fusiform Gyrus  Inferior Occipital Gyrus  Middle Occipital Gyrus  Middle Occipital Gyrus  Thalamus  Inferior Frontal Gyrus  Superior Frontal Gyrus  Superior Frontal Gyrus  Superior Frontal Gyrus  Precentral Gyrus  Precuneus  Superior Temporal Gyrus  Inferior Parietal Lobule  Middle Temporal Gyrus  Caudate  Fusiform Gyrus  Fusiform Gyrus  Posterior Cingulate Gyrus  Parahippocampal Gyrus  Medial Frontal Gyrus | | R  L  R  L  L  R  R  L  R  L  L  L  L  L  L  L  L  L  R  L  R  L | 17  17  18  18  19  19  45  9  8  6  6  22  40  21  20  6 | 5 -98 -1  -19 -99 -9  21 -86 -14  -35 -88 -14  -35 -84 9  34 -87 10  16 -21 12  -46 23 10  13 49 31  -13 51 38  -13 20 62  -36 -8 58  -5 -54 31  -52 13 -2  -52 -44 23  -55 -15 -6  -12 3 20  -36 -49 -16  34 -40 -17  -0 -58 27  34 -43 -10  -9 -5 58 | 5.14  4.37  3.51  3.52  4.26  3.37  3.68  5.97  4.27  4.53  4.99  3.76  7.45  5.24  6.82  5.00  4.51  3.11  3.92  3.52  4.89  8.93 |
| **Forgiveness and Unforgiveness > Rest** | | | | | |
| Anterior Cingulate  Anterior Cingulate  Superior Frontal Gyrus  Parahippocampal Gyrus  Middle Temporal Gyrus  Inferior Frontal Gyrus  Precuneus  Middle Cingulate  Inferior Parietal Lobule  Posterior Cingulate  Medial Frontal Gyrus  Superior Frontal Gyrus | | L  R  L  L  L  L  L  L  L  R  R  R | 10  46  10  6 | -6 18 22  3 12 22  -23 54 -1  -25 -43 4  -38 -47 8  -43 41 7  -0 -75 33  -0 -22 30  -38 -61 40  -9 -26 23  9 56 1  9 -1 63 | 4.70  3.24  4.45  5.06  6.77  3.34  3.02  3.02  2.89  2.73  3.32  4.35 |
| **Forgiveness and Unforgiveness > Pre-hurtful and Hurtful** | | | | | |
| Anterior Cingulate Cortex  Medial Cingulate Gyrus  Posterior Cingulate Gyrus  Precuneus  Inferior Parietal Lobule  Parahippocampal Gyrus  Superior Frontal Gyrus  Middle Frontal Gyrus  Middle Frontal Gyrus  Middle Frontal Gyrus  Caudate  Inferior Parietal Lobule  Superior Frontal Gyrus  Superior Temporal Gyrus  Middle Temporal Gyrus  Insula | R  R  R  R  R  L  L  R  R  L  L  L  L  L  L  L | | 7  40  19  8  6  10  10  40  11  13 | 9 31 18  9 -19 32  9 -34 22  9 -74 35  49 -45 50  -32 -42 -4  -2 18 50  29 -1 50  28 49 -3  -42 46 -3  -17 18 4  -48 -58 43  -22 45 -6  -39 9 -21  -39 -47 6  -40 11 -2 | 2.70  2.59  2.72  7.37  3.18  3.35  2.57  4.01  3.04  2.98  2.56  3.11  3.00  3.19  2.86  2.82 |
| **Hurtful > Pre-hurtful** | | | | | |
| Superior Frontal Gyrus  Middle Temporal Gyrus  Precuneus  Supramarginal Gyrus | | R  L  L  L | 22  7 | 9 3 66  -56 -31 4  -1 -69 37  -47 -54 32 | 4.12  11.69  4.56  5.45 |
| **Pre-Hurtful > Hurtful** | | | | | |
| Lingual Gyrus  Lingual Gyrus  Posterior Cingulate  Postcentral Gyrus  Precentral gyrus  Inferior Parietal Lobule  Anterior Cingulate | R  L  L  L  L  L  L | | 17  17  2  6  25 | 7 -95 -3  -14 -94 -3  -14 -56 17  44 -28 30  -46 -1 30  -49 -35 30  -1 10 -3 | 3.40  5.77  3.57  4.91  4.43  3.82  4.16 |
| **Forgiveness > Pre-Hurtful** | | | | | |
| Superior Frontal Gyrus  Anterior Cingulate Cortex  Superior Temporal Gyrus  Hippocampus  Precuneus  Insula  Cuneus | | R  R  L  L  R  L  R | 6  32  22  7  13 | 12 9 62  19 38 16  -45 -4 -6  -28 -42 7  8 -68 38  -41 6 -3  13 -70 30 | 4.62  7.52  5.25  6.44  6.26  4.61  6.10 |
| **Unforgiveness > Pre-Hurtful** | | | | | |
| Medial Frontal Gyrus  Inferior Parietal Lobule  Anterior Cingulate Gyrus | | L  L  L | 8  32 | -2 23 45  -55 -52 48  -5 22 26 | 4.15  5.06  6.96 |
| **Forgiveness > Hurtful** | | | | | |
| Cingulate Gyrus  Posterior Cingulate Gyrus  Parahippocampal Gyrus  Inferior Parietal Lobule  Insula | | L  L  L  R  R | 24  40 | -4 -15 35  -11 -32 32  -24 -44 -5  55 -47 45  29 23 4 | 6.26  8.03  5.24  5.55  6.77 |
| **Unforgiveness > Hurtful** | | | | | |
| Anterior Cingulate Gyrus | | L |  | -5 25 26 | 6.87 |
| **Forgiveness > Unforgiveness** | | | | | |
| Dorsolateral Prefrontal Cortex  Posterior Cingulate Cortex  Fusiform Gyrus  Parahippocampal Gyrus  Cuneus  Lingual Gyrus | | L  R  L  L  R  L | 18  18 | -44 15 51  16 -52 14  -35 -47 -12  -40 -14 -18  13 -75 14  -17 -69 0 | 5.99  5.45  4.35  5.87  8.27  9.15 |
