# Supplementary material for: How the brain heals emotional wounds: the functional neuroanatomy of forgiveness
Source: Front Hum Neurosci. 2013 Dec 9;7:839. doi: 10.3389/fnhum.2013.00839 (PMC3856773; doi:10.3389/fnhum.2013.00839)
Supplement: Supplementary file 3 [file 48075__Data_Sheet_2.DOCX]

**Supplementary** **Table 1B. Clusters of significant differences in neural responses for the conditions of interest. Brain areas have been described in their cluster distribution (cluster center of mass is reported) and volumes.**

| **Brain Area** | **HS** | **Coordinates** | | | **Cluster size** |
| --- | --- | --- | --- | --- | --- |
| **Forgiveness and Unforgiveness > Pre-hurtful and Hurtful** | | | | | |
| Middle Frontal | L | -36 | 45 | 8 | 1212 |
| Superior/Middle Frontal | R | 27 | 39 | 14 | 748 |
| Superior Frontal | R | 14 | -1 | 68 | 710 |
| Anterior Cingulate | R/L | -3 | 21 | 22 | 3755 |
| Medial/Posterior Cingulate | R/L | -1 | -25 | 30 | 2412 |
| Medial Cingulate | L | -21 | -26 | 36 | 558 |
| Middle Temporal | L | -33 | -44 | 3 | 1773 |
| Inferior Parietal | R | 44 | -56 | 40 | 1640 |
| Precuneus | R | 9 | 73 | 33 | 1318 |
| **Hurtful > Pre-hurtful** | | | | | |
| Superior Frontal | R | 9 | 16 | 64 | 1192 |
| Supramarginal | L | -50 | -56 | 39 | 961 |
| Precuneus | R | 3 | -65 | 39 | 763 |
| Middle Temporal | L | -59 | -32 | 2 | 601 |
| **Hurtful < Pre-hurtful** | | | | | |
| Middle Frontal | L | -38 | 47 | 6 | 841 |
| Precentral/Superior Frontal | L | -49 | -3 | 45 | 2406 |
|  | L | -32 | -15 | 61 | 1209 |
| Posterior Parietal | L | -30 | -79 | 30 | 2894 |
|  | L | -26 | -61 | 57 | 859 |
|  | R | 28 | -71 | 26 | 885 |
| Posterior Cingulate | L/R | -11 | -56 | 18 | 1065 |
| Supramarginal/Inferior Parietal | R | 51 | -25 | 30 | 1212 |
|  | L | -45 | -33 | 39 | 2259 |
| Medial Occipital | R | 14 | -86 | -4 | 4939 |
|  | L | -13 | -91 | -3 | 2387 |
|  | L | -32 | -75 | -11 | 515 |
| Caudate | L | -2 | 12 | 0 | 1323 |
| **Forgiveness > Hurtful** | | | | | |
| Medial Frontal | R | 21 | 30 | 6 | 2695 |
|  | L | -21 | 29 | 5 | 1080 |
| Inferior Frontal | R | 43 | 42 | 4 | 541 |
| Anterior Cingulate | L/R | -2 | 20 | 23 | 1150 |
| Posterior Cingulate | L/R | -2 | -26 | 31 | 2181 |
| Inferior Parietal | R | 50 | -54 | 45 | 1873 |
| Parahippocampal | L | -30 | -53 | 3 | 1038 |
| **Unforgiveness > Hurtful** | | | | | |
| Anterior Cingulate | L/R | -4 | 24 | 22 | 1149 |
|  |  |  |  |  |  |
| Inferior Parietal |  | 46 | -53 | 46 | 1103 |
| Posterior Cingulate | L/R | 1 | -20 | 31 | 535 |
| Inferior Frontal | L | -42 | 47 | 4 | 506 |
| **Forgiveness > Pre-Hurtful** | | | | | |
| Superior Frontal | R | 12 | 5 | 66 | 1047 |
| Insula | L | -41 | 2 | -8 | 1692 |
| Middle Frontal | R | 20 | 31 | 15 | 1410 |
| Cingulate | L/R | -1 | 4 | 21 | 3578 |
| Middle Temporal | L | -32 | -45 | 4 | 2477 |
| Superior/Inferior Parietal | L | -43 | -63 | 45 | 1975 |
|  | R | 45 | -60 | 40 | 1674 |
| Precuneus | R/L | 4 | -71 | 35 | 2283 |
| Cerebellum | L | -40 | -61 | -28 | 681 |
| **Unforgiveness > Pre-Hurtful** | | | | | |
| Anterior Cingulate | L/R | -4 | 21 | 27 | 1880 |
| Superior/Inferior Parietal | L | -23 | -63 | 45 | 1860 |
| Posterior Cingulate | L/R | -3 | -22 | 30 | 537 |
| **Forgiveness > Unforgiveness** | | | | | |
| Dorsolateral Prefrontal | L | -42 | 15 | 47 | 628 |
| Cuneus/Posterior Cingulate | R | 16 | -63 | 9 | 1731 |
|  | L | -16 | -64 | 8 | 1034 |
| Middle Temporal/Parahippocampal | R | 49 | -13 | -15 | 690 |
|  | L | -38 | -8 | -11 | 843 |
| Inferior Parietal | R | 54 | -47 | 42 | 514 |
